# CCK‐1 and CCK‐2 receptor agonism do not stimulate GLP‐1 and neurotensin secretion in the isolated perfused rat small intestine or GLP‐1 and PYY secretion in the rat colon

**DOI:** 10.14814/phy2.14352

**Published:** 2020-01-26

**Authors:** Ida M. Modvig, Charlotte B. Christiansen, Jens F. Rehfeld, Jens J. Holst, Simon Veedfald

**Affiliations:** ^1^ Department of Biomedical Sciences The Panum Institute Faculty of Health and Medical Sciences University of Copenhagen Copenhagen Denmark; ^2^ Translational Metabolic Physiology NNF Center for Basic Metabolic Research The Panum Institute Faculty of Health and Medical Sciences University of Copenhagen Copenhagen Denmark; ^3^ Department of Clinical Biochemistry Rigshospitalet Copenhagen Denmark

**Keywords:** bombesin, CCK, cholecystokinin, colon, ex vivo, gastrin, GIP, GLP‐1, glucagon‐like peptide‐1, glucose‐dependent insulinotropic polypeptide, hormones, isolated perfused colon, isolated perfused small intestine, neurotensin, peptide YY, PYY, rat, secretion, vasoactive intestinal peptide, VIP

## Abstract

Gastrin and cholecystokinin (CCK) are hormones released from endocrine cells in the antral stomach (gastrin), the duodenum, and the jejunum (CCK). Recent reports, based on secretion experiments in an enteroendocrine cell line (NCI‐H716) and gastrin receptor expression in proglucagon‐expressing cells from the rat colon, suggested that gastrin could be a regulator of glucagon‐like peptide‐1 (GLP‐1) secretion. To investigate these findings, we studied the acute effects of CCK‐8 (a CCK1/CCK2 (gastrin) receptor agonist) and gastrin‐17 (a CCK2(gastrin) receptor agonist) in robust ex vivo models: the isolated perfused rat small intestine and the isolated perfused rat colon. Small intestines from Wistar rats (*n* = 6), were perfused intraarterially over 80 min. During the perfusion, CCK (1 nmol/L) and gastrin (1 nmol/L) were infused over 10‐min periods separated by washout/baseline periods. Colons from Wistar rats (*n* = 6) were perfused intraarterially over 100 min. During the perfusion, CCK (1 nmol/L), vasoactive intestinal peptide (VIP) (10 nmol/L), and glucose‐dependent insulinotropic polypeptide (GIP) (1 nmol/L) were infused over 10‐min periods separated by washout/baseline periods. In the perfused rat small intestines neither CCK nor gastrin stimulated the release of GLP‐1 or neurotensin. In the perfused rat colon, neither CCK or VIP stimulated GLP‐1 or peptide YY (PYY) release, but GIP stimulated both GLP‐1 and PYY release. In both sets of experiments, bombesin, a gastrin‐releasing peptide analog, served as a positive control. Our findings do not support the suggestion that gastrin or CCK participate in the acute regulation of intestinal GLP‐1 secretion, but that GIP may play a role in the regulation of hormone secretion from the colon.

## Graphical abstract

Recent reports, based on secretion experiments in an enteroendocrine cell line (NCI‐H716) and gastrin receptor expression in proglucagon‐expressing cells from the rat colon, suggested that gastrin could be a regulator of glucagon‐like peptide‐1 (GLP‐1) secretion. To investigate these findings, we studied the acute effects of CCK‐8 (a CCK1/CCK2 (gastrin) receptor agonist) and gastrin‐17 (a CCK2(gastrin) receptor agonist) in the isolated perfused rat small intestine and the isolated perfused rat colon. Our findings do not support the suggestion that gastrin or CCK participate in the acute regulation of intestinal GLP‐1 secretion, but that GIP, unexpectedly, may play a role in the regulation of hormone secretion from the colon.

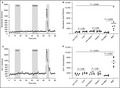

## 1 INTRODUCTION

Gastrin and cholecystokinin (CCK) are gastrointestinal hormones released from endocrine cells in the antral stomach (gastrin) and the duodenum and jejunum (CCK) (Rehfeld, 1998). CCK has also been identified in neurons innervating the small intestine and colon (Larsson & Rehfeld, 1979; Schultzberg et al., 1980).

Recently, it has been suggested, based on gastrin receptor expression in a human enteroendocrine cell line (NCI‐H716) and in proglucagon‐expressing cells harvested from rat colon tissue, that gastrin, released from the proximal gastrointestinal tract is involved in the regulation of the more distal gut hormone glucagon‐like peptide‐1 (GLP‐1) (Cao, Cao, & Liu, 2015). CCK‐2 (gastrin) receptors were found to be expressed in NCI‐H716 cells (mRNA, protein, and immunohistochemistry), a human colon epithelial cell line, and in rat colonic L‐cells (immunohistochemistry). In NCI‐H716 cells, the gastrin concentration necessary to elicit GLP‐1 release over 2 hr ranged between 10^–8^ and 10^–6^ M gastrin.

We are not aware of reports of the effects of gastrin on gut hormone secretions in vivo (humans or animals) or in ex vivo animal models. Meanwhile, gastrin‐releasing peptide and a non‐mammalian homolog bombesin, are powerful stimulants of gut hormone release (Roberge, Gronau, & Brubaker, 1996).

A potential role for CCK in the regulation of distal gut hormone secretion has previously been investigated using both in vivo and ex vivo models. Thus, in the isolated perfused rat small intestine (ileum), CCK‐8 at a dose of 50 pmol/L did not stimulate GLP‐1 or NT secretion (Dumoulin, Dakka, Plaisancie, Chayvialle, & Cuber, 1995). In the perfused isolated rat colon, the same CCK dose (50 pmol/L) increased PYY secretion (apparent after 15 min of perfusion), but increasing the dose to 250 pmol/L did not further increase PYY secretion (Plaisancié, Bernard, Bernard, Chayvialle, & Cuber, 1995). In the same study, intraarterial GIP increased PYY secretion, while VIP did not. In subsequent experiments, employing the isolated rat colon, CCK infusion was associated with increased PYY secretion (Ko et al., 2011) and increased colonic motility.

Incubation of fetal rat intestinal cell cultures with CCK (10^–12^ to 10^–6^ M) did not increase GLP‐1 concentrations in the medium (Brubaker & Brubaker, 1991) and infusion of CCK (16 ng bolus and 25 ng/h) into anesthetized rats did not stimulate the secretion of GLP‐1 (Roberge & Brubaker, 1991). Meanwhile, in isolated perfused porcine small intestine, CCK at a concentration of 1 nmol/L (~100–500 times normal postprandial plasma concentrations) increased GLP‐1 concentrations in the venous effluent, whereas, lower (0.1 nmol/L) and higher (10 nmol/L) concentrations of CCK did not stimulate GLP‐1 secretion (Hansen & Holst, 2002).

In human studies evaluating the effect of CCK infusions on gastrointestinal hormone release (Brennan et al., 2007; Rohde et al., 2016), it was not possible to discriminate between direct effects of CCK on gut endocrine cells and indirect effects elicited by, for example, CCK‐stimulated bile release, but CCK infusions, giving rise to near complete gallbladder emptying, were associated with increased plasma GLP‐1 (Rohde et al., 2016). Bile acids should be considered as a potential mediator as they stimulate gut hormone secretions ex vivo (Brighton et al., 2015; Christiansen et al., 2019; Kuhre et al., 2018; Plaisancié, Dumoulin, Chayvialle, & Cuber, 1995) and in humans under physiologically relevant conditions (Meyer‐Gerspach et al., 2013; Nielsen et al., 2017; Wu et al., 2013).

To investigate the recent findings regarding gastrin‐stimulated GLP‐1 secretion(Cao et al., 2015), we tested whether gastrin and CCK at supra‐physiological levels (the same concentrations that was reported to stimulate GLP‐1 secretion in porcine intestines) would elicit secretion of GLP‐1, PYY, and neurotensin, gut peptides released from different enteroendocrine cell subpopulations (L‐cells (GLP‐1 and PYY, small intestine and colon) and N‐cells (neurotensin, distal small intestine), respectively). We included CCK recognizing that CCK at high concentrations is able to activate not only the CCK‐1 receptor but also the gastrin (CCK‐2) receptor due to structural similarities between gastrin and CCK. In the colon experiments, we included vasoactive intestinal peptide (VIP) and glucose‐dependent insulinotropic polypeptide (GIP) because these peptides have been suggested to be hormone secretagogues in the colon (Plaisancié, Bernard, et al., 1995). For our experiments, we relied on isolated perfused preparations of rat small intestine and colon, previously demonstrated to respond adequately to known stimuli of gut hormone secretion.

## 2 METHODS

### 2.1 Animals

Studies were performed with permission from the Danish Animal Experiments Inspectorate and the local animal studies regulatory body in accordance with the guidelines of the Danish legislation governing animal experimentation (1987).

Male Wistar rats (small intestinal perfusions, mean (*SD*) = 277 (17) g), colon perfusions (265 (11) g), were obtained from Janvier labs (Le Genest‐Saint‐Isle, housed 2–4 rats per cage and kept on a 12:12 hr light/dark cycle with ad libitum access to standard chow and water in the animal house at the Faculty of Health and Medical Sciences, The University of Copenhagen.

### 2.2 Intestinal perfusions

On the experimental day, animals (non‐fasted) were anesthetized with a subcutaneous Hypnorm/Midazolam injection (doses 0.0158 mg fentanyl citrate + 0.5 mg fluanisone + 0.25 mg midazolam/100 g) and placed on a heating plate (37°C).

#### 2.2.1 Small intestinal perfusions

The abdomen was opened and the entire large intestine and the distal half of the small intestine carefully removed, leaving 30(2) cm of the upper small intestine in situ. A plastic tube was inserted into the oral opening of the small intestinal lumen and the intestinal luminal content was gently removed with isotonic saline (room temperature). The small intestinal lumen was perfused with saline at a steady flow of 0.25 ml/min. A catheter was placed in the superior mesenteric artery and the intestine was perfused at a rate of 7.5 ml/min with a gassed perfusion buffer (95% O_2_/5% CO_2_) heated to 37°C. (Modvig, Kuhre, & Holst, 2019).

#### 2.2.2 Colon perfusions

The abdomen was opened and the colon was isolated by ligating the vessels to the small intestine, cecum, spleen, stomach, kidneys as well as the celiac artery. This allowed the vascular isolation of 10.8 (0.3) cm of the colon, corresponding to a segment spanning from the most proximal part of the colon to the part just proximal to the entry of the inferior mesenteric artery. A plastic tube was inserted into the oral opening of the colonic lumen and the luminal content gently removed with isotonic saline (room temperature). The colonic lumen was perfused with saline at a steady flow of 0.15 ml/min. A catheter was inserted into the abdominal aorta, which was ligated proximally and distally to the outlet of the superior mesenteric artery, and perfusion was started at a rate of 3 ml/min with a gassed perfusion buffer (95% O_2_/5% CO_2_) heated to 37°C (Christiansen et al., 2018, 2019).

When perfusion had been established, animals were sacrificed by cardiac perforation and the preparation allowed to equilibrate for ~30 min before samples were collected. The effluent perfusion buffer was collected over 1‐min periods from a portal vein catheter into low adsorbent tubes (minisorb, Nunc), which were placed immediately into ice and then stored at −20°C until analyses.

### 2.3 Perfusion buffer

The buffer consisted of a Krebs‐Ringer bicarbonate buffer supplemented with 0.1% BSA (albumin fraction V; Merck, cat. no. 1.12018.0500, Ballerup, Denmark), 3.5 mmol/L glucose, 5% dextran T‐70 (to balance oncotic pressure; Pharmacosmos, Holbaek, Denmark), 10 µmol/L 3‐Isobutyl‐1‐methylxanthine (IBMX) (Sigma‐Aldrich, cat. no. 5,879), 2 ml/L of Vamin (cat. no. 11338; Fresenius Kabi, Uppsala, Sweden), and 5 mmol/L pyruvate, fumarate, and glutamate. pH was adjusted with hydrochloric acid to 7.4–7.5.

### 2.4 Peptide infusions

CCK‐8, gastrin‐17 (provided by JF Rehfeld), VIP (Bachem cat.no. H‐3775), and rat GIP (provided by Mette Rosenkilde) were dissolved in water with 1% human serum albumin, and aliquots were prepared for each experiment. On the day of experiments, vials were thawed and infusions prepared by diluting the peptide aliquots with the perfusion buffer described above. Bombesin (BBS) (Bachem, cat.no. H‐2155) was dissolved in water with 1% human serum albumin and further diluted in perfusion buffer to reach a final perfusate concentration of 10 nmol/L.

### 2.5 Peptide hormone analyses

Gut hormones were measured using in‐house radioimmunoassays: total GLP‐1 was measured using anti‐serum no. 89390, directed against the C‐terminus of GLP‐1 (Orskov, Rabenhoj, Wettergren, Kofod, & Holst, 1994). Total Neurotensin was measured using antiserum no 3D97 (Kuhre, Bechmann, Wewer Albrechtsen, Hartmann, & Holst, 2015). Total PYY immunoreactivity (PYY_1–36_ + PYY_3–36_) was measured with a porcine antiserum (cat. no T‐4093; Bachem) (Toräng, Veedfald, Rosenkilde, Hartmann, & Holst, 2015).

### 2.6 Statistics

Hormone responses (fmol/L × min) were determined as the areas under the curve (AUC) for the 10‐min preceding and during infusions (5 min for BBS infusions) using the trapezoidal rule. Total hormone outputs were calculated by multiplying hormone responses with the perfusion flow rates—small intestine perfusions (0.0075 L/min) and colon perfusions (0.003 L/min). If repeated measurements (RM) one‐way analysis of variance (ANOVA) resulted in significant differences (*p* < .05), the pre‐stimulus and during stimulus outputs were compared with Bonferroni corrections (for small intestinal perfusions (pre CCK vs. CCK, pre gastrin vs. gastrin, pre BBS vs. BBS) and for colon perfusions (pre CCK vs. CCK, pre VIP vs. VIP, pre GIP vs. GIP). Data are means with standard errors of the means (*SEM*) unless otherwise stated. All statistical analyses were performed with GraphPad Prism 8.0 for Mac (San Diego, CA, USA).

## 3 RESULTS

### 3.1 Small intestinal perfusions

**Figure 1:**
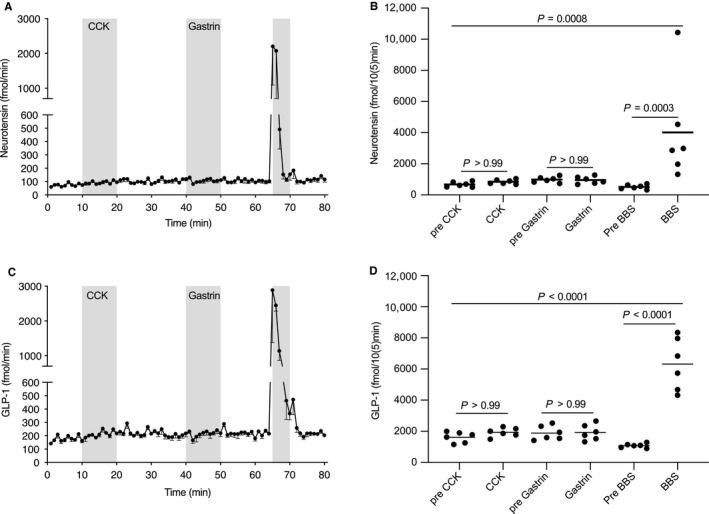
Neurotensin (a) and Glucagon‐like peptide‐1 (GLP‐1) (c) output (fmol/min) in the venous effluent from the isolated perfused rat small intestine (Wistar, *n* = 6). After a 10‐min baseline, cholecystokinin (CCK) was infused intraarterially (1 nmol/L, 10 min). After a 20‐min washout, gastrin was infused intraarterially (1 nmol/L, 10 min). Finally, bombesin (BBS, a gastrin‐releasing peptide analog) was infused over 5 min as a positive control. Hormone outputs) for neurotensin (b) and GLP‐1 (D). Filled circles represent areas hormone output over 10 min for individual rats, (5 min periods for BBS). Data are means with standard errors of the mean. Post hoc comparisons were made by one‐way analyses of variance (ANOVA) with Bonferroni corrections

NT concentrations over the course of the experiment were analyzed by one‐way ANOVA (*p* = .0008) (Figure 1a and b). This result allowed post hoc comparisons. Neither CCK nor gastrin increased neurotensin secretion (*p* = .99), but BBS did (*p* = .003) (Figure 1b).

GLP‐1 concentrations over the course of the experiment were analyzed by one‐way ANOVA (*p* = .0001) (Figure 1c and d), which allowed post hoc comparisons. Neither CCK nor gastrin increased neurotensin secretion (*p* = .99), but BBS did (*p* < .0001) (Figure 1d).

### 3.2 Colon perfusions

**Figure 2:**
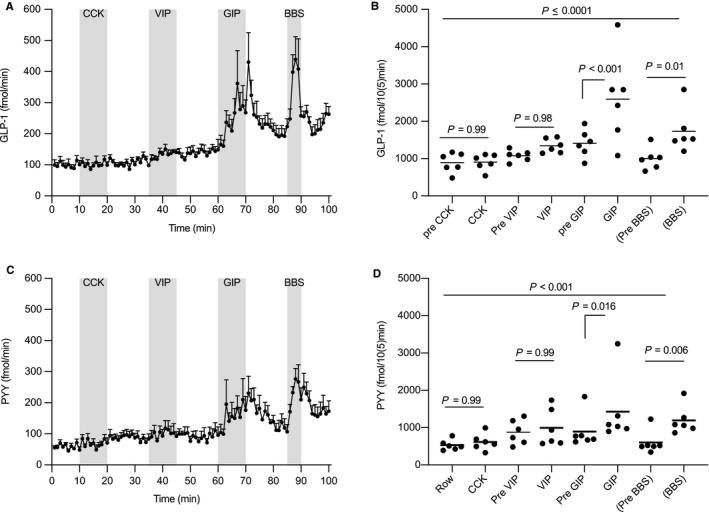
Glucagon‐like peptide‐1 (GLP‐1) (a) and peptide YY (PYY) (c) output (fmol/min) in the venous effluent from the isolated perfused rat colon (Wistar, *n* = 6). After a 10‐min baseline, Cholecystokinin (CCK) was infused intraarterially (1 nmol/L, 10 min). After a 15‐min washout, vasoactive intestinal peptide (VIP) was infused intraarterially (10 nmol/L, 10 min), after a 15‐min washout glucose‐dependent insulinotropic polypeptide (GIP) was infused intraarterially (1 nmol/L, 10 min). Finally, Bombesin (BBS, a gastrin‐releasing peptide analog) was infused over 5 min as a positive control. Hormone outputs for GLP‐1 (b) and PYY‐1 (d). Filled circles hormone outputs over 10‐min periods for individual rats, (5‐min periods for BBS). Data are means with standard errors of the mean. Post hoc comparisons were made by one‐way analyses of variance (ANOVA) with Bonferroni corrections

GLP‐1 concentrations over the course of the experiments were analyzed by one‐way ANOVA (*p* = .011) (Figure 2a and b). This result allowed post hoc comparisons. CCK did not increase GLP‐1 secretion (*p* = .99). Neither did VIP (*p* = .98). However, GIP (*p* < .001) and BBS (*p* = .01) increased GLP‐1 secretion (Figure 2b).

PYY concentrations over the course of the experiments were analyzed by one‐way ANOVA ( *p* = .001) (Figure 2c and d). This result allowed post hoc comparisons. Neither CCK nor VIP increased PYY secretion (*p* = .99). Both GIP (*p* = .016) and BBS stimulated PYY release (*p* = .006) (Figure 2d).

Neurotensin concentrations were also analyzed, but concentrations were all lower than the detection limit (1–2 pmol/L) even during bombesin stimulations (data not shown).

## 4 DISCUSSION

We did not find any stimulatory effect of high concentrations of CCK or gastrin on the release of GLP‐1 and neurotensin from the rat small intestine. Neither could we find any stimulatory effect of CCK and VIP on GLP‐1 and PYY secretion from the rat colon. However, GIP stimulated GLP‐1 and PYY release from the perfused rat colon.

The possibility that hormones released from the proximal gut could participate in the regulation of gut hormones secreted from the distal gut has been extensively investigated in animal models with particular focus given to the potential effect of CCK.

The motivation for this study was the finding that gastrin (CCK2) receptors had been identified in a human enteroendocrine cancer cell line (NCI‐H716) and that gastrin given at high (10–1,000 nmol/L) supraphysiologic doses increased GLP‐1 concentrations in the medium of an enteroendocrine cancer cell line over 2 hr. Gastrin receptors were also reported to be located on GLP‐1 producing cells from the rat colon, but secretion experiments using tissue from the colon were not performed (Cao et al., 2015).

In earlier studies, CCK has been reported to stimulate GLP‐1 secretion (Hansen & Holst, 2002; Ko et al., 2011; Plaisancié, Bernard, et al., 1995). However, in two of these studies the stimulatory effects of CCK were modest and not reproducible with higher CCK doses.

Intraarterial VIP has previously been tested in the isolated perfused rat small intestinal model, and in that intestinal segment VIP weakly elicited GLP‐1 secretion (Herrmann‐Rinke, Vöge, Hess, & Göke, 1995). In our experiments, VIP did not elicit GLP‐1 or PYY secretion from the isolated perfused colon.

Intravenous administration of GIP in rats has been reported to elicit GLP‐1 secretion, albeit the rise in plasma GLP‐1 was not apparent until 20 min after the initiation of the GIP administration (Roberge & Brubaker, 1993). In the perfused rat ileum, intraarterial GIP has also been reported to stimulate GLP‐1 secretion from the isolated perfused ileum (Herrmann‐Rinke et al., 1995). We find that also in the colon intraarterial GIP stimulates GLP‐1 and PYY secretion.

Our study has limitations. We did not use doses higher than 1 nmol/L of CCK, gastrin, and GIP (10 nmol/L of VIP was used to emulate its release from enteric nerves). Our study aimed to determine whether gastrin and CCK would impact GLP‐1 and neurotensin secretion via the endocrine pathway under physiological conditions. To be sure not to overlook an effect we employed supraphysiological concentrations. As all the stimuli employed are peptides (not small molecules) acting via G‐protein coupled receptors, we do not expect off target effects—at the modestly supraphysiological doses employed. We did not evaluate the bioactivity of the peptides employed independently from the perfusion studies, but all peptides were procured from trusted sources, their purity and structural integrity were controlled in our laboratories (CCK and gastrin) or by the manufacturers (GIP, VIP, BBS*)* and were prepared fresh for each perfusion, which would limit any degradation to minimal spontaneous hydrolysis. Plasma CCK concentrations would under normal circumstances be about 50–200 times lower than the employed dose. Peak plasma gastrin concentrations after liquid and solid meals would be expected to be about 20 pmol/L (Sonne, Rehfeld, Holst, Vilsbøll, & Knop, 2014) and 20–40 pmol/L, respectively (Hornnes et al., 1980; Rasmussen et al., 1997), that is, ~25–50 times lower than the dose used in our experiments. During treatment with proton pump inhibitors gastrin concentrations increase ~2–4 fold, but even under these circumstances the gastrin concentration used in our perfusions would still be about 8–10 fold higher than circulating concentration of gastrin (Obszynska et al., 2010; Rasmussen et al., 1997).

We used a GIP dose only 10–15 fold higher than what would be observed in individuals with rapid gastric emptying and Roux‐en‐Y gastric bypass (Jørgensen et al., 2013; Plamboeck et al., 2013). Thus, given the effect of GIP observed in the perfused colon it cannot be ruled out that GIP might be able to stimulate colonic gut hormone secretions in vivo.

## 5 CONCLUSION

We find that (1) Acute CCK1/CCK2 receptor agonism does not stimulate GLP‐1 or neurotensin secretion from isolated perfused rat small intestine and (2) Acute CCK1/CCK2 receptor agonism does not stimulate GLP‐1 and PYY secretion from the isolated perfused rat colon.

## AUTHOR CONTRIBUTIONS

All authors participated in the design of the study. IMM performed small intestinal perfusions. CBC performed colon perfusions. SV, CBC performed GLP‐1, PYY, and neurotensin analyses. SV analyzed the data. All authors interpreted the data. SV wrote the manuscript. IMM, CBC, JFR, and JJH critically reviewed and revised the manuscript. All authors approved the publication of this final version of the manuscript.
